# Relationship between Mastery Motivation and Sensory Processing Difficulties in South Korean Children with Developmental Coordination Disorder

**DOI:** 10.1155/2020/6485453

**Published:** 2020-01-02

**Authors:** Hee Young Kim

**Affiliations:** Department of Occupational Therapy, Honam University, 62399, Republic of Korea

## Abstract

The aim of this study was to identify the correlation between mastery motivation and sensory processing difficulties among South Korean children with developmental coordination disorder (DCD). Ninety-nine children aged 4–7 years with DCD participated. The Dimensions of Mastery Questionnaire was used to assess the mastery motivation of the children, and the Short Sensory Profile was used to assess the children's sensory processing difficulties. All subjects showed lower mastery motivation and definite differences in sensory processing. Mastery motivation was significantly correlated with sensory processing (*r* = −0.34, *p* = 0.01). Mastery motivation predicted 41.1% of the sensory processing. In particular, a negative reaction to failure in mastery situations scale (*p* < 0.01) and general competence compared to peers scale (*p* < 0.05) in mastery motivation were significant predictors. This study indicated that sensory processing difficulties and lack of mastery motivation were identified among children with DCD in South Korea. And the children with high mastery motivation show less difficulty in sensory processing. It is suggested to develop possible solution for higher mastery motivation to improve sensory processing of the children with DCD in South Korea.

## 1. Introduction

The American Psychiatric Association (2013) diagnoses these children who have remarkably reduced coordination abilities, which lead to difficulties in daily life and poor academic achievement compared to typically developed children with the same chronological age, as having developmental coordination disorder (DCD) [1]. In developmental coordination disorder (DCD), impairments in developmental motor skills lead to difficulties in performing everyday activities. These challenges affect the ability to perform the skillful movements required for activities of daily living, including studying, self-care, and performance of tasks [2].

Recent studies have demonstrated that difficulties in sensory processing and integration, as well as in motor skills, may influence how children with DCD interact with their environments [2–4]. Children with DCD have been found to have lower function in visual perception, visual motor skills, and motor planning [4]. And DCD children show greater tactile sensitivity [5] and movement sensitivity and lower energy level and underresponsiveness and sensation seeking than normal children [6].

The function of sensory processing and integration is to facilitate the interpretation of sensory input from one's body and the environment, leading to a meaningful and appropriate behavioral response [7]. Impaired sensory processing may lead to misperception of the environment, restricted individualization, and distortion of reality [8], and it also has a negative impact on children's participation in school life and social interactions [9]. These issues, in turn, may lower self-confidence and limit the ability to identify feedback on behavior [10].

In sensory integration theory, Ayres and Tickle described motivation as the desire or willingness to respond to a stimulus that has been registered or to ignore it. And internal motivation to seek out, experience, and master challenges is often lacking in the child with sensory integrative dysfunction [11]. They additionally identified poor “inner drive” and environment/body percept as contributing to motivation impairments but did not delineate how these components interacted. Therefore, the motivation in the SI theory is treated to be consistent with the definition of other researchers [12, 13]. Similar to the concept of inner drive in sensory integration theory, Vlachou and Farrell [14] conceptualized mastery motivation as a force that impels the child to engage in the process of the task in an effort to master it regardless of whether the task is completed [10].

Mastery motivation is a psychological force that stimulates a person to make focused, persistent, and independent attempts to solve a problem, acquire a skill, or complete a task that poses a moderate or strong challenge [15]. Mastery motivation emerges in late infancy, and it may be a precursor to self-determination, setting a course of increasing independence, developing a growing sense of competence, and an enhanced perception of one's ability to control one's environment [16]. If children experience pleasure in persistence and mastering tasks, their mastery motivation increases and they seek additional opportunities to engage in challenging tasks, which facilitates the development of new knowledge and skills [17]. Highly motivated children are more likely than children with low levels of mastery motivation to learn successful strategies, which foster children's competence in problem solving [18]. Therefore, it can be inferred that highly motivated children have different sensory integration experiences and more opportunities to mature them.

Many studies have indicated that DCD children have low motivations [19, 20]. Engel and Hannah reported that children with DCD as young as 5 years expressed limited self-efficacy and it affects the motivation to engage in social and psychical activities [21]. Another study identified that reduced motivation for physical activity in DCD children reduces opportunities for exercise and fitness development [22]. This can be inferred that the low motivation of DCD children reduces participation in activities and limits opportunities for sensory integration. In South Korea, there are some studies on DCD [23, 24], but none of them have been conducted on the basic of sensory processing and motivational characteristics. Research on DCD children, especially in the preschool age, is very scarce.

Therefore, the first purpose of this study was to investigate the difficulties in sensory processing and mastery motivation among South Korean children with DCD. The second objective was to explore the relationship between mastery motivation and sensory processing difficulty. The last goal was to investigate the impact of mastery motivation on sensory processing in children.

## 2. Methods

### 2.1. Participants

The population of children between ages four and seven years in the geographical area of trust was estimated to be 56,313 [25]. The reported prevalence of DCD is 5-6% [1]. At the higher rate of incidence, the expected number of children with DCD aged four to seven years would be as many as 3,378. The required sample size, calculated with the Raosoft power calculator [26], was 94 (95% confidence interval and 10% margin of error).

The study participants were 99 South Korean children aged 4–7 years who had DCD and attended a special day care center and rehabilitation clinic. All of the children were diagnosed with DCD by a pediatrician or developmental neurologist. DCD diagnosis was established based on 4 criteria described in the *Diagnostic and Statistical Manual of Mental Disorders* (fifth edition) [1]: total Movement Assessment Battery for Children Second Edition (MABC-2) [27] cutoff score of ≤5%; Developmental Coordination Disorder Questionnaire 2007 (DCDQ'07) score < 46 [28], as indicated in a parental report describing the level of ADL coordination of the child; and no physical or neurological deficits, as reported by the parents and confirmed based on health records.

The subjects (99) included 58 (58.6%) boys and 41 (40.6%) girls. Among these subjects, 43 (43.4%) were aged 48–59 months, 23 (23.2%) were aged 60–71 months, and 33 (33.4%) were aged 72–84 months (see Table 1).

### 2.2. Procedure

Occupational therapists assessed the children's sensory processing level using the Short Sensory Profile (SSP) and mastery motivation using the Dimensions of Mastery Questionnaire (DMQ).

### 2.3. Measures

#### 2.3.1. Short Sensory Profile

This instrument is a shorter version of the Sensory Profile, an instrument used for evaluating sensory processing abilities. The SSP is based on the report of a child's main caregiver. It is comprised of 38 items that demonstrated the highest discriminative power of atypical sensory processing among all of the items from the longer version of the Sensory Profile. The seven sections of the SSP found in a normative sample are tactile sensitivity, test/smell sensitivity, movement sensitivity, underresponsive/seeks sensation, auditory filtering, low energy/weak, and visual/auditory sensitivity. The internal consistency of the sections within the scale ranged from 0.70 to 0.90 [29]. The internal validity correlations for the sections ranged from 0.25 to 0.76 and were all significant at p95% for identifying children with and without sensory modulation difficulties [29]. Items are scored on a five-point scale. Both section scores and a total score are recorded on the SSP. The possible range of raw scores on the total scale is 38–190, with higher scores (155–190) reflecting normal performance. A score of 142–154 reflects a probable difference in performance, and a score of 38–141 reflects a definite difference in performance [29].

#### 2.3.2. Dimensions of Mastery Questionnaire

The DMQ assesses several aspects of adults' and children's perceptions of children's mastery-related behaviors. The DMQ is one of several measurement techniques, including challenging structured tasks and semistructured play, developed to assess mastery motivation [15]. DMQ 17 has 45 Likert-type items, each rated from 1 to 5 (from not at all typical to very typical), and seven scales as follows. Four scales for the instrumental (persistence) aspects of mastery motivations are (1) the object-oriented persistence scale (called persistence at cognitive tasks for school-age children and teens, 9 items), (2) the gross motor persistence scale (8 items), (3) the social persistence/mastery motivation with adults scale (6 items), and (4) the social persistence/mastery motivation with children scale (6 items). Two scales for the expressive aspects of mastery motivation are (5) the mastery pleasure scale, which measures the positive affect after finishing a task and/or while working on a task (6 items), and (6) the negative reaction to failure in mastery situations scale (5 items). Finally, one scale to assess competence or the ability to master tasks, in contrast to the motivation to master tasks, is (7) the general competence compared to peers scale (5 items). Each of the first five scales includes one negatively worded item that is reverse coded when computing the scale scores. The negative reaction to failure items are all worded in the same direction, and negative reactions (upset, avoid, etc.) are scored as 5. The competence scale has 2 out of 5 items worded negatively and reverse coded. Morgan et al. [30] showed that the DMQ 17 has acceptable to good internal consistency (alphas > 0.74), and Jozsa and Molnar [31] reported test-retest reliability ranging from 0.61 to 0.94.

### 2.4. Data Analysis

All data were analyzed using IBM SPSS statistical version 20.0 (SPSS Inc., Chicago, IL, USA). To test the first question (Do children with DCD have sensory processing difficulties?), a descriptive test was selected. To test question 2 (Do children with DCD have mastery motivation difficulties?), a descriptive test was selected. To test question 3 (Is there a relationship between reported mastery motivation difficulties and sensory processing difficulties?), Pearson's correlation test was used. To test question 4 (Does mastery motivation affect sensory processing?), multiple linear regression was used. A significance level of *p* < 0.05 was established.

## 3. Results

### 3.1. Mastery Motivation of Subjects

The total score of mastery motivation was 122.09. The object-oriented persistence scale score was 21.73, the social persistence/mastery motivation with adults scale score was 16.86, the social persistence/mastery motivation with children scale score was 16.02, the mastery pleasure scale score was 19.85, the negative reaction to failure in mastery situations scale score was 13.32, and the general competence compared to peers scale score was 12.69 (see Table 2).

### 3.2. Sensory Profile of Subjects

All members of the sample group (100%) showed a definite difference in sensory processing. Among the children, 99 (99.0%) showed a definite difference in tactile sensitivity. In taste/smell sensitivity, 53 (53.5%) of the children showed a definite difference, 28 (28.3%) of the children showed typical performance, and 18 (18.2%) of the children showed a probable difference. In movement sensitivity, 89 (89.9%) of the children showed a definite difference, 6 (6.1%) of the children showed a probable difference, and 4 (4.0%) of the children showed a probable difference. In underresponsive/ seeks sensation, 88 (88.9%) of the children showed a definite difference, 6 (6.1%) of the children showed a probable difference, and 5 (5.1%) of the children showed typical performance. In auditory filtering, 97 (97.7%) of the children showed a definite difference. In low energy/weak, 69 (69.7%) of the children showed a definite difference, 20 (20.2%) of the children showed typical performance, and 10 (10.1%) of the children showed a probable difference. All members of the sample group (100%) showed a definite difference in visual/auditory sensitivity (Table 3).

### 3.3. Relationship between Mastery Motivation and Sensory Processing

When examining the correlations between mastery motivation and the sensory profile, mastery motivation was significantly correlated with tactile sensitivity (*r* = –0.24, *p* = 0.05), auditory filtering (*r* = −0.26, *p* = 0.05), low energy/weak (*r* = –0.34, *p* = 0.01), and sensory processing (*r* = −0.34, *p* = 0.01). Sensory processing was significantly correlated with the object-oriented persistence scale (*r* = −0.36, *p* = 0.01), gross motor persistence scale (*r* = −0.35, *p* = 0.01), social persistence/mastery motivation with adults scale (*r* = −0.45, *p* = 0.01), mastery pleasure scale (*r* = −0.22, *p* = 0.01), negative reaction to failure in mastery situations scale (*r* = 0.51, *p* = 0.01), and general competence compared to peers scale (*r* = −0.48, *p* = 0.01) (Table 4).

### 3.4. Mastery Motivation to Predict Sensory Processing

To investigate the effect of mastery motivation on sensory processing, multiple linear regression analysis was performed for the full sample. The outcome variable for the model was the SSP total score, and the predictor variables were the object-oriented persistence scale, gross motor persistence scale, social persistence/mastery motivation with adults scale, social persistence/mastery motivation with children scale, mastery pleasure scale, negative reaction to failure in mastery situations scale, and general competence compared to peers scale. The model was significant at *p* < 0.001, and it explained 41.1% of the variance in the SSP total score. The negative reaction to failure in mastery situation scale (*p* < 0.01) and the general competence compared to peers scale (*p* < 0.05) were significant predictors (Table 5).

## 4. Conclusions

This study was conducted to assess mastery motivation and sensory processing, to investigate the relationship between mastery motivation and sensory processing, and to examine the effect of mastery motivation on sensory processing in South Korean children with DCD.

We used the Short Sensory Profile questionnaire, which measures children's responses to sensory events in everyday life. All samples in this study showed a definite difference in the total score of the SSP. Allen and Casey [2] found that 88% of children with DCD aged 5-12 years showed differences in sensory processing and integration, including 32% with definite differences and 56% with some differences. Engel and Shochat [32] reported that 73% to 87% of children with DCD exhibited impairment in visual control, visuospatial processing, and visuomotor sensory integration. In the present study, the number of children showing sensory processing difficulties was greater than the number reported by the previous study, which is likely to be because our study participants were receiving rehabilitation at a special day care center and rehabilitation clinic and the sensory processing assessment was probably affected by their parents' perspectives.

In this study, the mean score for mastery motivation in children with DCD was 19.1, which is very low—only 1.1 points higher than the minimal total score of 18 [33]. The result is consistent with those of other studies reporting a lack of mastery motivation in children with DCD [34, 35].

The children with low mastery motivation tended to have low tactile sensitivity, difficulties in auditory filtering, “low energy/weak,” and sensory processing difficulties. Mastery motivation had a statistically significant effect on sensory processing in children with DCD (*p* < 0.01), with 41.1% explanatory power. Children with DCD have lower perceptions of their own physical competence [36], a passive lifestyle because of negative attitudes toward physical activity, and fewer opportunities to engage in physical activity due to lower motivation [22]. Lower participation in activities leads to less experience in sensory processing. In particular, experience is likely to be more limited in activities that involve uncomfortable or challenging sensory processing.

Elements of mastery motivation had a significant influence on sensory processing. This is especially the case for negative reaction to failure in mastery situations (*β* = 0.471, *p* < 0.001) and general competence compared to peers (*β* = −0.329, *p* < 0.01). This finding suggests that sensory processing is affected by attitudes toward, and perceptions of, activities. Children tend to avoid activities when they have little expectation of success or anticipate feeling mentally exhausted, i.e., frustrated or embarrassed [37]. Sufficient mastery motivation improves children's self-confidence, a sense of personal achievement, and participation in leisure activities, and it affects their life satisfaction and sense of well-being [38] by helping them interact with their environment and learn from these interactions [39]. In particular, children who experience permanent disability and lack motivation may develop symptoms such as secondary unrealistic attitude, anxiety, a sense of inferiority, and a lack of cooperation with family, which, in turn, may limit their physical activity [40]. Moreover, these children become unable to take full advantage of their abilities, even when they have the skills necessary to perform tasks [41]. The lack of motivation in children with DCD leads to a passive lifestyle by reducing their opportunities for physical activity and their development of motor skills [22]. Therefore, the reduction of failures, provisioning of successful experiences, and alleviation of psychological stress due to failures, to lower negative thoughts about activities, are likely to improve motivation and sensory processing.

The results of this study offer partial support for the inner drive component of the sensory integration (SI) approach and the theory of mastery motivation. The SI approach is often used for children with sensory processing difficulties. The theory suggests play activities and sensory-enhanced interaction as strategies for encouraging an adaptive response in children. In addition, the theory delineates activities that help children engage with sensory processing and with motor and planning skills [7]. Inner drive is an important part of the therapeutic process. It allows children to seek and engage with sensory motor activities spontaneously and, as a result, to experience a new external environment and acquire sensory input due to their changed behavior [42]. Along with the inner drive component of the SI theory, motivation, as a psychological force behind persistent efforts to overcome the environment and become competent, is likely to have had a major impact on sensory processing function in this study by enabling children to overcome sensory activities that were uncomfortable and difficult to handle [43].

The theory of mastery motivation posits that children with a high expectation of success develop preferences for sensory activities by choosing what they can do and what they feel is worthwhile. The theory suggests that children choose activities for which they are motivated and that engaging with activities offers children the opportunity to gain experience and become mature [33]. On the other hand, children become easily frustrated by sensory challenges encountered during activities, and they become excluded from activities when they lack motivation.

This study has particular significance as the first study to investigate the mastery motivation and sensory processing difficulties and to examine sensory processing skills based on mastery motivation in South Korea children with DCD. The results of the study have limited generalizability, as study participants were not recruited and sampled randomly. In addition, the assessment of mastery motivation was based on parent reports. A multidimensional assessment that includes both parent reports and investigator observations would improve accuracy and comprehensiveness.

The limit of this study was the failure to investigate dyslexia in children with DCD. According to the Canadian census, 23% of children had DCD symptoms and 22% of them had both DCD and dyslexia [44]. Children with both DCD and dyslexia have aggression and low self-esteem [45, 46], so this may be related to the mastery motivation of this study. Further research is needed taking into account dyslexia in DCD children.

In conclusion, sensory processing difficulties and lack of mastery motivation were identified among children with DCD in South Korea. And the children with high mastery motivation show fewer difficulties in sensory processing. Understanding the effects of mastery motivation on sensory processing in children with DCD is an important part of efforts to improve sensory processing. In particular, it is recommended to enhance DCD children's sensory processing through improved attitudes and perceptions of activities, such as a negative reaction to failure and comparison of abilities with those of peers.

## Figures and Tables

**Table 1:** Subjects of the study (*N* = 99).

Variable		Number (%)
Gender	Male	58 (58.6)
	Female	41 (40.6)

Age (months)	48–59	43 (43.4)
	60–71	23 (23.2)
	72–84	33 (33.4)

**Table 2:** Mastery motivation of subjects (*N* = 99).

Variable	*M* ± SD
Object-oriented persistence scale	2.41 ± 0.83
Gross motor persistence scale	2.70 ± 1.00
Social persistence/mastery motivation with adults scale	2.81 ± 0.53
Social persistence/mastery motivation with children scale	2.67 ± 0.94
Mastery pleasure scale	3.31 ± 0.98
Negative reaction to failure in mastery situations scale	2.66 ± 0.84
General competence compared to peers scale	2.54 ± 0.88
Mastery motivation	19.1 ± 6.00

**Table 3:** Sensory profile of subjects (*N* = 99).

Variable	*M* ± SD	*N* (%)		
		Typical performance	Probable difference	Definite difference
Tactile sensitivity	12.48 ± 3.93	0 (0.0)	1 (1.0)	99 (99.0)
Taste/smell sensitivity	10.41 ± 4.94	28 (28.3)	18 (18.2)	53 (53.5)
Movement sensitivity	5.82 ± 3.15	4 (4.0)	6 (6.1)	89 (89.9)
Underresponsive/seeks sensation	16.95 ± 5.79	5 (5.1)	6 (6.1)	88 (88.9)
Auditory filtering	14.84 ± 5.71	0 (0.0)	2 (2.1)	97 (97.9)
Low energy/weak	19.00 ± 7.15	20 (20.2)	10 (10.1)	69 (69.7)
Visual/auditory sensitivity	10.50 ± 4.12	3 (3.0)	10 (10.1)	86 (86.9)
Total	89.95 ± 22.85	0 (0.0)	0 (0.0)	100 (100.0)

**Table 4:** Relationships between mastery motivation and sensory processing (*N* = 99).

	Object-oriented persistence scale	Gross motor persistence scale	Social persistence/mastery motivation with adults scale	Social persistence/mastery motivation with children scale	Mastery pleasure scale	Negative reaction to failure in mastery situations scale	General competence compared to peers scale	Mastery motivation
Tactile sensitivity	−0.21^∗^	−0.19	−0.26^∗^	−0.31^∗∗^	−0.18	0.38^∗∗^	−0.38^∗∗^	−0.24^∗^
Taste/smell sensitivity	−0.14	−0.11	−0.06	−0.25^∗^	0.01	0.29^∗∗^	−0.26^∗^	−0.12
Movement sensitivity	−0.18	−0.22^∗^	0.02	−0.27^∗^	−0.04	0.48^∗∗^	−0.33^∗∗^	−0.15
Underresponsive/seeks sensation	−0.19	−0.06	0.05	−0.24^∗^	−0.03	0.49^∗∗^	−0.26^∗^	−0.08
Auditory filtering	−0.31^∗∗^	−0.22^∗^	0.02	−0.40^∗∗^	−0.19	0.38^∗∗^	−0.40^∗∗^	−0.26^∗^
Low energy/weak	−0.30^∗∗^	−0.52^∗∗^	−0.16	−0.22^∗^	−0.43^∗∗^	0.09	−0.33^∗∗^	−0.41^∗∗^
Visual/auditory sensitivity	−0.26^∗^	−0.20	−0.14	−0.39^∗∗^	0.01	0.41^∗∗^	−0.21^∗^	−0.19
Sensory processing	−0.36^∗∗^	−0.35^∗∗^	−0.11	−0.45^∗∗^	−0.22^∗^	0.51^∗∗^	−0.48^∗∗^	−0.34^∗∗^

^∗^ *p* < 0.05, ^∗∗^*p* < 0.01.

**Table 5:** Mastery motivation to predict sensory processing (*N* = 99).

Variables	Unstandardized coefficients		Standardized coefficients	*t*	*p*	Collinearity statistics	
	*B*	SE	Beta			Tolerance	VIF
(Constant)	87.486	11.977		7.304	0.001		
Object-oriented persistence scale	0.531	0.493	0.174	1.076	0.285	0.229	4.374
Gross motor persistence scale	-0.051	0.437	-0.018	-0.117	0.907	0.256	3.900
Social persistence/mastery motivation with adults scale	0.292	0.623	0.041	0.468	0.641	0.792	1.262
Social persistence/mastery motivation with children scale	-0.646	0.437	-0.159	-1.477	0.143	0.520	1.922
Mastery pleasure scale	-0.762	0.517	-0.195	-1.475	0.144	0.343	2.913
Negative reaction to failure in mastery situations scale	2.570	0.484	0.471	5.306	0.001	0.762	1.313
General competence compared to peers scale	-1.705	0.682	-0.329	-2.499	0.014	0.346	2.889

*R* ^2^ = 0.453, adjusted *R*^2^ = 0.411.

## Data Availability

The data used to support the findings of this study are restricted in order to protect subjects.

## References

[1] American Psychiatric Association (2013). *Diagnostic and Statistical Manual of Mental Disorders*.

[2] Allen S., Casey J. (2017). Developmental coordination disorders and sensory processing and integration: incidence, associations and co-morbidities. *The British Journal of Occupational Therapy*.

[3] Zoia S., Castiello U., Blason L., Scabar A. (2005). Reaching in children with and without developmental coordination disorder under normal and perturbed vision. *Developmental Neuropsychology*.

[4] Goyen T. A., Lui K., Hummell J. (2011). Sensorimotor skills associated with motor dysfunction in children born extremely preterm. *Early Human Development*.

[5] Loh P. R., Piek J. P., Barrett N. C. (2011). Comorbid ADHD and DCD: examining cognitive functions using the WISC-IV. *Research in Developmental Disabilities*.

[6] Zwicker J. G., Missiuna C., Harris S. R., Boyd L. A. (2010). Brain activation of children with developmental coordination disorder is different than peers. *Pediatrics*.

[7] Ayres A. J., Robbins J. (1979). *Sensory Integration and the Child*.

[8] Jadidi J., Mirshoja M. S. (2016). The impact of the sensory integration approach on positive and negative symptoms in a patient with non-paranoid schizophrenia: a case report. *Middle East Journal of Rehabilitation and Health*.

[9] Dunn W., Little L., Dean E., Robertson S., Evans B. (2016). The state of the science on sensory factors and their impact on daily life for Children. *OTJR: Occupation, Participation and Health*.

[10] Miller E., Miller-Kuhaneck H. (2006). The relationship among sensory preferences, play preferences, motivation, and mastery in guiding children’s play: a review of the literature, part 2. *American Occupational Therapy Association’s Sensory Integration Special Interest Section Quarterly*.

[11] Ayres A. J., Tickle L. S. (1980). Hyper-responsivity to touch and vestibular stimuli as a predictor of positive response to sensory integration procedures by autistic children. *American Journal of Occupational Therapy*.

[12] Koegel R. L., Mentis M. (1985). Motivation in childhood autism: can they or won’t they?. *Journal of Child Psychology and Psychiatry*.

[13] Dawson G., Webb S. J., McPartland J. (2005). Understanding the nature of face processing impairment in autism: insights from behavioral and electrophysiological studies. *Developmental Neuropsychology*.

[14] Vlachou M., Farrell P. (2000). Object mastery Motivation in Pre-school children with and without disabilities. *Educational Psychology*.

[15] Morgan G. A., Harmon R. J., Maslin-Cole C. A. (1990). Mastery motivation: definition and measurement. *Early Education and Development*.

[16] Shazia H., Chua B. S., Murnizam H., Carmella E. A. Mastery motivation and cognitive development among 18-36 month old toddlers: a developmental perspective.

[17] Bronson M. B. (2000). *Self-Regulation in Early Childhood: Nature and Nurture*.

[18] Barrett K. C., Morgan G. A., MacTurk R. H., Morgan G. A. (1995). Continuities and discontinuities in mastery motivation during infancy and toddlerhood: a conceptualization and review. *Advances in Applied Developmental Psychology, 12. Mastery Motivation: Origins, Conceptualizations, and Applications*.

[19] Dunford C., Missiuna C., Street E., Sibert J. (2005). Children’s perceptions of the impact of developmental coordination disorder on activities of daily living. *British Journal of Occupational Therapy*.

[20] Rodger S., Watter P., Marinac J., Woodyatt G., Ziviani J., Ozanne A. (2007). Assessment of children with developmental coordination disorder (DCD): motor, functional, self efficacy and communication abilities. *New Zealand Journal of Physiotherapy*.

[21] Engel-Yeger B., Hanna Kasis A. (2010). The relationship between Developmental Co-ordination Disorders, child's perceived self-efficacy and preference to participate in daily activities. *Child: Care, Health and Development*.

[22] Katartzi E. S., Vlachopoulos S. P. (2011). Motivating children with developmental coordination disorder in school physical education: The self-determination theory approach. *Research in Developmental Disabilities*.

[23] Nam S. M., Lee K. J., KIM S. J., Lee Y. H., Kwon Y. H., KIM M. J. (2017). A preliminary study of reaction time in Korean children with developmental coordination. *The Korean Journal of Elementary Physical Education*.

[24] Kim M. J. (2017). A basic study of prevalence and characteristics of motor coordination among children with developmental coordination disorder in Korea. *The Korean Journal of Elementary Physical Education*.

[25] Korea Statistical Information Service (2017). Population and housing census/resident registration population. http://kosis.kr/easyViewStatis/customStatisIndex.do?vwcd=MT_TM1_TITLE&menuId=M_03_01.

[26] Inc R. (2004). Sample size calculator. http://www.raosoft.com/samplesize.html.

[27] Henderson S. E., Sugden D. A., Barnett A. L. (2007). *Movement Assessment Battery for Children-2: Movement ABC-2: Examiner’s Manual*.

[28] Wilson B., Kaplan B., Crawford S., Roberts G. (2007). Developmental Coordination Questionnaire 2007 (DCDQ’07). https://dcdq.ca/index.php.

[29] McIntosh D. N., Miller L. J., Shyu V., Dunn V., Dunn W. (1999). Overview of the Short Sensory Profile (SSP). *The Sensory Profile: Examiner’s Manual*.

[30] Morgan G. A., Wang J., Liao H. F., Xu Q., Barrett K. C., Fox N. A., Morgan G. A., Fidler D. J., Daunhauer L. A. (2013). Using the Dimensions of Mastery Questionnaire to assess mastery motivation of English- and Chinese-speaking children: psychometrics and implications for self-regulation. *Handbook of Self-Regulatory Processes in Development: New Directions and International Perspectives*.

[31] Jozsa K., Molnar E. D., Barrett K. C., Fox N. A., Fidler DJ M. G. A., Daunhauer L. A. (2013). The relationship between mastery motivation, self regulated learning and school success: a Hungarian and European perspective. *Handbook on Self Regulatory Processes in Development: New Directions and International Perspectives*.

[32] Engel-Yeger B., Shochat T. (2012). The relationship between sensory processing patterns and sleep quality in healthy adults. *Canadian Journal of Occupational Therapy*.

[33] Dichter-Blancher T. B., Busch-Rossnagel N. A., Knauf-Jensen D. E. (1997). Mastery motivation: appropriate tasks for toddlers. *Infant Behavior and Development*.

[34] Macnab J. J., Miller L. T., Polatajko H. J. (2001). The search for subtypes of DCD: is cluster analysis the answer?. *Human Movement Science*.

[35] Visser J. (2003). Developmental coordination disorder: a review of research on subtypes and comorbidities. *Human Movement Science*.

[36] Cantell M., Kooistra L. (2002). *Long-Term Outcomes of Developmental Coordination Disorder*.

[37] Watkinson E. J., Dwyer S. A., Nielsen A. B. (2005). Children theorize about reasons for recess engagement: does expectancy-value theory apply?. *Adapted Physical Activity Quarterly*.

[38] Shikako-Thomas K., Shevell M., Schmitz N. (2013). Determinants of participation in leisure activities among adolescents with cerebral palsy. *Research in Developmental Disabilities*.

[39] Glenn S., Dayus B., Cunningham C., Horgan M. (2007). Mastery motivation in children with Down syndrome. *Down Syndrome Research and Practice*.

[40] Buffart L. M., Westendorp T., van den Berg-Emons R. J., Stam H. J., Roebroeck M. E. (2009). Perceived barriers to and facilitators of physical activity in young adults with childhood-onset physical disabilities. *Journal of Rehabilitation Medicine*.

[41] Kim Y. J., Ahn S. H. (2014). Types of Motivation in Young Children : Associations with Young Children's Temperament and Their Mothers' Interactions. *Korean Journal of Child Studies*.

[42] Bundy A. C., Lane S., Murray E. A., Fisher A. G. (2002). *Sensory Integration : Theory and Practice*.

[43] Tyffany K. (2012). *Effectiveness of sensory integration and behavioral interventions on nonengagement in preschool aged children*.

[44] Kirby A., Sugden D. A., Chambers M. E. (2005). Overlapping conditions: overlapping management: services for individuals with developmental coordination disorder. *Children with Developmental Coordination Disorder*.

[45] Piek J. P., Barrett N. C., Allen L. S. R., Jones A., Louise M. (2005). The relationship between bullying and self-worth in children with movement coordination problems. *British Journal of Educational Psychology*.

[46] Skinner R. A., Piek J. P. (2001). Psychosocial implications of poor motor coordination in children and adolescents. *Human Movement Science*.

